# Impact of Systemic Delays for Patient Access to Oncology Drugs on Clinical, Economic, and Quality of Life Outcomes in Canada: A Call to Action

**DOI:** 10.3390/curroncol31030110

**Published:** 2024-03-11

**Authors:** Sandeep Sehdev, Joanna Gotfrit, Martine Elias, Barry D. Stein

**Affiliations:** 1 The Ottawa Hospital Cancer Centre, Ottawa, ON K1H 8L6, Canada; ssehdev@toh.ca (S.S.); jgotfrit@toh.ca (J.G.); 2 Myeloma Canada, Dorval, QC H9P 2V4, Canada; melias@myeloma.ca; 3 Colorectal Cancer Canada, Montreal, QC H3Z 2P9, Canada

**Keywords:** cancer, oncology, access, funding, drugs, reimbursement, health technology assessment

## Abstract

Canada has one of the most complex and rigorous drug approval and public reimbursement processes and is, unfortunately, one of the countries with the longest delays in drug access. To assess the overall impact of systemic delays in access to cancer therapy, a targeted literature review (TLR) was performed to identify studies associated with the clinical, economic, and quality of life impacts of delayed access to oncology drugs. Using MEDLINE/PubMed databases and snowballing, four unique records met the eligibility criteria. Results revealed that clinical outcomes were the most impacted by systemic delays in access to oncology drugs (e.g., life years lost, overall survival, and progression-free survival). The four articles retrieved by the TLR specifically illustrated that a substantial number of life years could potentially be saved by increasing systemic efficiency regarding the development, approval, and reimbursement processes of new drugs for advanced malignancies. It is imperative that initiatives are put in place to improve the performance and speed of Canadian drug regulatory and health technology assessment (HTA) processes, especially for new cancer therapeutics. The proposed solutions in this paper include better coordination between HTA and Canadian payers to harmonize coverage decisions, international collaborations, information sharing, and national standards for timeliness in oncology drug access.

## 1. Introduction

In Canada, cancer has a major impact on society and remains the leading cause of death. Recent estimates suggest that cancer is responsible for 28.2% of deaths nationwide and that two out of five Canadians will be diagnosed with cancer in their lifetime [1]. Although major advances have been made in oncology treatment, the number of new cancer cases continues to increase due to the growth and aging of the population. Indeed, an estimated 233,900 Canadians were diagnosed with cancer in 2022, resulting in approximately 85,100 deaths. Furthermore, the current 5-year net survival is estimated to be 64% for all cancers combined [1].

Canada has one of the most complex and rigorous drug approval and public reimbursement processes. Specifically, drugs are marketed after having demonstrated efficacy and safety through clinical trials and receiving Health Canada’s regulatory approval. A federal agency called the Patented Medicines Prices Review Board (PMPRB) ensures that the prices of patented drugs are not excessive, based on specified regulations [2]. Then, a health technology assessment (HTA) provides an evidence-based funding recommendation to Canadian provinces and territories. Except for Quebec, which receives recommendations from the *Institut national excellence en santé et services sociaux* (INESSS) [3], all provinces receive guidance from the Canadian Agency for Drugs and Technologies in Health (CADTH) for oncology and non-oncology drugs [4]. While recommendations are not fully binding, they are followed very closely by provinces and territories. After receiving a positive recommendation from CADTH or INESSS, which is generally conditional on a price reduction and specific reimbursement criteria, the pan-Canadian Pharmaceutical Alliance (pCPA) negotiates pricing (for public reimbursement) with manufacturers for most drugs, including cancer treatments [5]. Finally, each province or territory makes an independent decision on whether to publicly fund the drug, considering the CADTH’s recommendation as well as their mandates, policies, jurisdictional priorities, and budget. If price negotiations come to a successful agreement, a Letter of Intent (LOI) is created. The latter enumerates the terms and conditions for funding a drug, which are further used to create a product listing agreement (PLA) between the manufacturer and the jurisdictions. These steps have evolved over more than 30 years, bolting new processes on instead of integrating them effectively.

Consequently, patient access to innovative drugs is usually greatly delayed due to the sequential federal and provincial processes in regulatory and reimbursement reviews. Such lengthy timelines, which can vary greatly across provinces and territories [6,7,8], may have a significant impact on patients’ survival and quality of life (QoL). This is especially true for cancer patients, in both the metastatic setting (given their reduced or limited life expectancy) and the curative setting (where adjuvant medicines improve the chance of cure) [9,10]. Indeed, a treatment delay as short as four weeks can increase cancer-related mortality [9]. QoL is also affected detrimentally by delayed access to new treatments, which could better control disease and improve, prevent, or delay serious cancer symptoms. Time is of the essence for our patients. Delays in access are especially tragic considering the increasing effectiveness of new breakthrough therapeutics, which are extending patients’ lives, but remain inaccessible to patients in need. 

Unfortunately, Canada is one of the countries with the longest delays in drug access, with a median time from marketing authorization to public reimbursement for oncology drugs of 581 days, compared to 382 days for 20 comparable countries [11]. In addition, delays to approval for any medicines are nearly twice as long in Canada relative to the median timelines of these same countries, while also funding fewer medications [11]. Canada is third from the bottom in a list of OECD nations with respect to the timeliness of access to new cancer drugs. All our approval and funding mechanisms could benefit from more efficient and timely processes, as recently achieved by other jurisdictions through innovative early managed access programs, such as the pioneering German early benefit assessment program [12].

To assess the overall impact of systemic delayed access to cancer therapy, a targeted literature review (TLR) was performed to identify studies associated with the clinical, economic, and patient QoL impacts of systemic delayed access to oncology drugs. Based on findings from that TLR, there is clearly a need for an urgent call to action to improve timely access to oncology drugs in Canada.

## 2. Materials and Methods

A comprehensive TLR was conducted by a single assessor. The Medical Literature Analysis and Retrieval System Online (MEDLINE) database was searched for the period spanning from 1 January 2017 to 21 July 2022 (the last date of the search). The MEDLINE database includes records of biomedical literature by accessing the following sources: MEDLINE Publisher, In-Data-Review, In-Process, and PubMed-not-MEDLINE records from the National Library of Medicine (NLM). Due to the possible delay between the date of online publication and the date of indexation in bibliographic databases, the PubMed database was also consulted for the year preceding the search (2021–2022). 

The filters used to identify studies were combined with search terms related to the PICOS framework (i.e., the population of interest, intervention, comparator, and outcomes). More specifically, studies including and limited to adult patients (≥18 years old) diagnosed with cancer and taking any cancer drugs were considered. Only articles reporting systemic delays (delays due to approval or reimbursement processes) were accepted, while articles reporting only individual delays (e.g., time from cancer diagnosis to the start of treatment already accessible) were excluded. Outcomes regarding clinical (survival rates or overall survival (OS), progression-free survival (PFS), disease-free survival (DFS), recurrence-free survival (RFS), and mortality), economic (health care costs, quality-adjusted life years (QALYs), and hospitalization), and QoL (surveys/questionnaires and patient-reported outcomes (PROs)) were included. The complete list of search terms for each outcome is presented in Supplementary Materials (Tables S1–S3). To be as comprehensive as possible, additional publications were identified by manually searching reference lists of the retrieved publications and prior literature reviews and meta-analyses, which is referred to as “snowballing.” 

The iterative searches were restricted to articles written in English or French, while also including ahead-of-print, in-process, and nonindexed records. Also, since this TLR aimed at reinforcing the need to limit the delay in treatment access in Canada, studies conducted in countries presenting comparable healthcare systems were included, such as the United States, Europe, the United Kingdom, and Australia. 

The management of records obtained through the search was conducted using EndNote, version X20. Duplicates found across the search on both databases were identified using automated procedures, which were then followed by a manual review to ensure the complete removal of those duplicates. The study selection process was performed by a single review and was based on a two-step approach: (i) titles/abstracts screening and (ii) in-depth review of full-text articles. Reasons for exclusion were documented at this stage. Finally, one reviewer extracted information from each included study using a predefined extraction form. The selection of articles through the different phases of the literature search was described using a Preferred Reporting Items for Systematic Reviews and Meta-Analyses (PRISMA) flow chart.

## 3. Results

### Search Results

**Figure 1:**
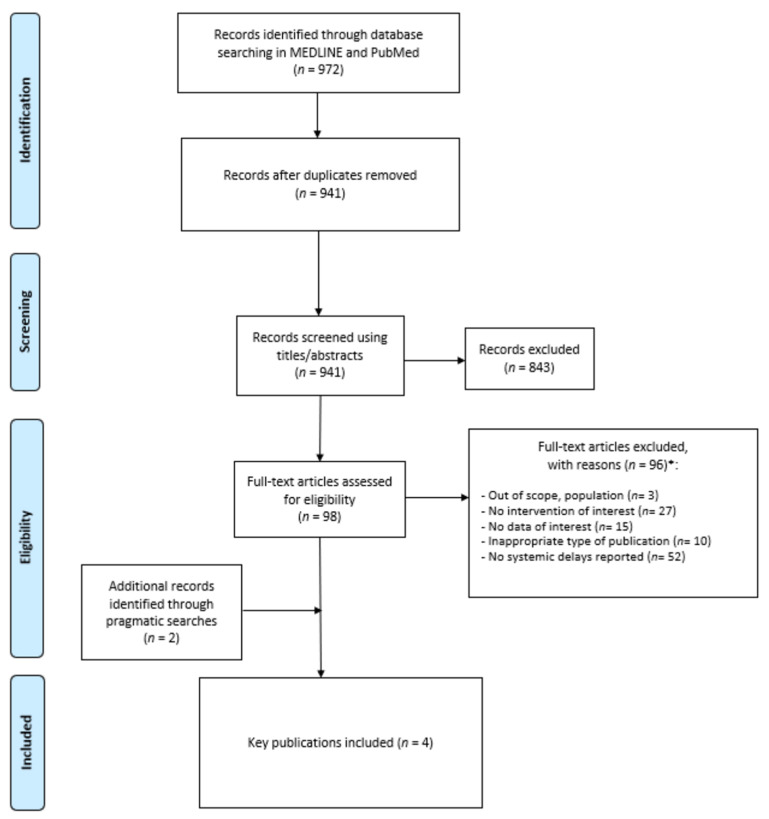
PRISMA flow chart. * More than one reason of exclusion could be reported.

Using the MEDLINE/PubMed databases, a total of 972 articles were identified. Of those, 31 duplicates were found, and 843 records were excluded based on titles and abstracts, leading to 98 articles being fully assessed. Of those, 96 were excluded. Snowballing methods yielded two additional records, and therefore, four unique records were included in the TLR [13,14,15,16]. The study selection process is depicted in the PRISMA flow chart (Figure 1). The most prevalent reason for exclusion was because of the types of delays. Indeed, most delays identified in the TLR were individual delays (*n* = 52). Study characteristics of the four retained publications are described in Table 1. Details on the objective, study design, types of cancer, types of treatment (i.e., chemotherapy, surgery, or radiation), and outcomes reported (i.e., OS, mortality, costs, etc.) are described.

The four articles reporting systemic delays were a retrospective cohort study [15], a public data analysis [13,14] and an analysis of literature and empirical modelling [16]. Multiple types of cancer were assessed in most studies [15], while the study by Vanderpuye-Orgle only assessed non-small cell lung cancer (NSCLC) [16]. Two studies were from Canada [13,14], while the other two studies were from multiple countries [15,16]. Also, even though the data were heterogeneous, the great majority of studies reported the impact of delayed access to treatment on clinical outcomes, more specifically live years lost (*n* = 2), life years saved (*n* = 1), OS (*n* = 1), and PFS (*n* = 1).

Finally, the types of treatments included in the articles were new therapies for metastatic cancers, chemotherapies, and targeted therapies.

The results of this TLR highlighted the outcomes impacted by delayed access to oncology drugs, the majority of which were clinical outcomes (e.g., life years lost, OS, and PFS). It is conceivable that delays associated with the lengthy drug approval and reimbursement processes will have an even greater impact. This was specifically analyzed in the four articles included in this review [13,14,15,16].

In the first study by Stewart and colleagues, the incremental improvement in median survival was multiplied by the number of patients in North America and worldwide dying annually from the relevant malignancy, which was then multiplied by the estimated time from drug discovery until approval [13]. The global median number of life years lost between the time of drug discovery and approval was 1,020,900. The authors concluded that, had the time required for patients to access drugs from discovery to approval been set at 5 years, the median number of life years saved would have reached 523,890 worldwide.

In a second study, the objective of Gotfrit and colleagues was to quantify the potential life years lost during the drug approval and funding process in Canada [14]. Drugs for advanced lung, breast, and colorectal cancer that underwent the HTA process between 2011 and 2016 were analyzed. Life years lost were calculated by multiplying documented OS and PFS improvements, the number of eligible patients, and the time from proof of efficacy (POE) to first public funding. Results demonstrated that the time from POE to first public funding ranged from 14.0 to 99.2 months (median 26.6 months). In total, the overall and progression-free life years lost from POE to first public funding were 39,067 (lung 32,367; breast 6691) and 48,037 (lung 9139, breast 15,827, colorectal 23,071), respectively. The study also highlighted the inequity of access across the country, where time intervals from first to last provincial funding ranged from 2.8 to 23.8 months (median 6.2 months).

In the study by Uyl-de Groot, a retrospective database analysis was conducted, with the authors aiming to assess the access in Europe to newly registered cancer drugs and to obtain more insight on the implications of these variations for patients [15]. Twelve cancer drugs were analysed in 28 European countries between 2011 and 2018, and outcomes assessed were time to patient access, speed of drug uptake, and potential loss of life years due to a delay in access. The analysis showed that the delay in patient access to ipilimumab (melanoma) and abiraterone (prostate cancer) has led to a potential loss of more than 30,000 life years.

Finally, the impact of delays on NSLSC patients was assessed in the study by Vanderpuye-Orgle. In this analysis, authors measured the durations and outcomes of regulatory and reimbursement reviews of NSCLC drugs in Canada and reference countries, the delays in Canada’s review for three NSCLC drugs, and the clinical and economic impact of these delays [16]. The drugs evaluated were nivolumab, afatinib, and pemetrexed. The analysis demonstrated that in Canada, reviews of NSCLC drugs took a medium of 216 days, and delays for the three drugs evaluated ranged between 5 and 94 days at Health Canada, 0 and 80 days at CADTH/pCODR, and 12 and 797 days in Canadian provinces. Consequently, 6400 patients may have been affected, totalling a loss of up to 1740 person years of life. In addition, this article has allowed us to evaluate the economic impact of systemic delayed access for NSLSC patients. Indeed, authors have estimated that the loss of 1740 person years of life and 1122 quality-adjusted life years was valued at over CAD112 million, which is only for NSLSC.

These examples illustrate that a substantial number of life years could potentially be saved by increasing the systemic efficiency regarding the development, approval, and reimbursement processes of new drugs for advanced malignancies. Initiatives should be put in place to improve and accelerate new oncology drug regulatory and funding processes, in the best interest of Canadian patients. Given the relative paucity of real-world data on the impact of regulatory delays, further evidence on this issue across jurisdictions is needed to determine the cost-effectiveness of our current processes.

## 4. Discussion

### 4.1. Call to Action

Our TLR reviewed studies assessing the systemic impact of delayed access to cancer treatment on different types of outcomes (clinical, economic, and quality of life). It was demonstrated that clinical outcomes, including OS and PFS, were the most affected by delayed access to oncology drugs. The review allowed us to retrieve four studies in which the impact of delayed access to oncology medicines due to systemic delays was assessed, demonstrating the great impact of lengthy government approval and reimbursement processes on patients’ outcomes, mainly potential life years lost.

There are significant disparities to access to new oncology drugs between Canada and other countries. Indeed, Canada’s public reimbursement process has worsened compared to its international peers, since Canada is the only country whose public reimbursement timelines saw a significant increase between 2010–2015 and 2011–2016 [17]. A report concluded that only 59% of cancer medicines were covered by public drug plans across provinces, comprising at least 80% of the eligible national public drug plan population, ranking Canada 17th out of 20 countries for oncology drug access [18]. Furthermore, in a recent publication by Skinner (2023), authors examined government performance regarding access to innovative cancer medicines and compared marketing authorizations and formulary listings in publicly funded drug plans in Canada, Germany, and the United States. Results showed that in Canada, an average of 11% of new cancer drugs approved for marketing from 2016 to 2020 in at least one of the three jurisdictions evaluated were listed on a public formulary by December 2021, compared to 73% and 90% for Germany and the United States, respectively. This deplorable reality significantly impacts Canadian patients and their families waiting to access the latest and most promising treatments, especially for cancer patients for whom treatment options may be limited or curative and for whom timing is critical.

These disparities between Canada and other countries are partly attributable to complex, overlapping sequential multi-step regulatory and funding processes involving federal, provincial, and private authorities (Health Canada, PMPRB, HTA organizations (CADTH and INESSS), pCPA, and different provincial and private payers) [19]. One study investigated the determinants of cancer drug funding decisions and timelines in Canada [20]. Drugs for advanced lung, breast, colorectal, melanoma, and neuroendocrine cancers undergoing the funding decision process from 2011 to 2019 were analyzed. Results indicated that determinants of drug funding included cancer type (neuroendocrine tumours had the shortest time-to-funding and were funded in at least 1 province 100% of the time, while drugs for colorectal cancer took the longest to fund and were only funded 29% of the time), drug class (immunotherapies had the shortest time-to-funding, whereas targeted therapies took the longest to fund), and pCODR recommendation (89% of drugs that received a positive pCODR recommendation were funded in at least one province, and all drugs with a negative recommendation were not funded in any province). The list price was not predictive of drug funding or the time to funding. It should be noted that the publicly available list price is not the final price paid by provinces (which is confidentially negotiated), and negotiated rebates often go back into general government coffers rather than into Ministries of Health. Consequently, the resulting benefits to the health care system may not be evident.

International regulatory collaborations between Canada and other countries exist, such as Project Orbis, an international partnership designed to give cancer patients faster access to promising cancer treatments [21,22]. Canada has joined the U.S. Food and Drug Administration and other international regulators to collaborate in such joint drug reviews for cancer, rare diseases, and conditions with limited treatment options, allowing for simultaneous review and earlier approval of these medications. However, such initiatives appear insufficient to overcome systemic delays, as only 28% of drugs approved by Health Canada qualified for faster regulatory review from these programs, compared to 82% in the United States [23].

Thus, there is a clear need for optimization of the approval and reimbursement process of oncology drugs to ensure timely and accelerated treatment access for cancer patients in Canada, which could be achieved with the solutions presented below.

### 4.2. Potential Solutions

In terms of access to innovative cancer treatments, short- and mid-term solutions are possible and should be rapidly implemented to improve the situation in Canada. Better coordination between HTA, pCPA, and provinces (e.g., concurrent reviews instead of sequential reviews) would result in timeliness in reimbursement nationwide. The implementation of a multi-provincial formulary approval process, such as a national pharmacare program, instead of a provincial system would also simplify the process and allow for greater consistency in reimbursement across Canada. The creation of the Canadian Drug Agency (CDA) is a first step towards making Canada’s drug system more sustainable and efficient [24]. Indeed, the CDA, which will be built from the existing CADTH and in partnership with provinces and territories, will be involved in the reduction of drug system duplication and lack of coordination.

Early benefit assessment programs, such as the successful AMNOG process in Germany, could be implemented [12]. Indeed, since 2011, Germany’s government has allowed a free pricing period at launch for the first 6 months (a period of 12 months before October 2022) of marketing for medicines with a new active substance. During that free-price period, all drugs must undergo a systematic assessment of their effectiveness compared to the standard of care, followed by a price negotiation based on this assessment. This patient-centered approach is recognized worldwide and, in part, explains why Germany is one of the fastest-growing European countries for oncology drug access [15]. The effect of AMNOG on launch delays was assessed by Bussgen (2023) [25]. The authors obtained launch data on pharmaceuticals for 30 European countries from the IQVIA database and used difference-in-difference (DiD) models. The results demonstrated that the introduction of AMNOG consistently reduced the magnitude of the decrease in launch delay in Germany compared to the comparator countries (staggered DiD: +4.31 months, *p* = 0.05). Furthermore, in the study by Uyl-de Groot previously described the average time to market in Europe amounted to 398 days (range 17–1187 days). The study reported that patients in Germany, the United Kingdom, and Austria had the most rapid access, with averages of 17, 22 and 31 days, respectively [15].

Furthermore, increased international collaborations to avoid duplication of efforts should be implemented, such as the Project Orbis model for Health Canada regulatory approval acceleration [21,22]. Project Orbis partners work together on the review of submissions for cancer drugs. Health Canada has been a partner in Project Orbis since its inception in May 2019. These collaborations should go beyond market approval and should be pursued for HTA processes. Indeed, health technology developers often must submit the same information, data, and analyses to different jurisdictions and at various points in time. This duplication of workload can constitute a significant and unnecessary administrative and cost burden and might contribute to delayed access to medicines. To overcome this challenge, the European HTA Regulation was created in December 2011, with the objective of harmonizing methodological standards and promoting collaboration among European HTA bodies [26]. This initiative will ensure that any evidence required for clinical assessment will be submitted only once by HTA developers. The European HTA regulation will be adopted in a stepwise manner, starting in 2025 onwards, where all new cancer medicines and advanced therapy medicinal products will be commonly assessed, while the final HTA appraisals and subsequent reimbursement decisions remain within the responsibility of each jurisdiction. From 2028 on, orphan medicinal products will also be included in the mutual clinical assessment. This promising initiative will allow a more efficient assessment of the most innovative medicines. Another example is the BeneluxA initiative, which comprises five countries (Belgium, the Netherlands, Luxembourg, Austria, and Ireland) and has the objective of improving access to innovative medicines at an affordable cost [27,28]. The different activities of this initiative include information sharing (re-use of HTA reports) and collaboration with mutual recognition of national HTA assessments. Examples of collaborations include expert referencing, where one HTA body shares an expert review with another HTA body, and mutual recognition of Assessments. These different inspiring international partnerships demonstrate approaches that can be taken to improve timely and appropriate patient access to oncology drugs.

The Australian HTA Reform aims to reduce the time to treatment access for Australian patients and to increase the commercial attractiveness of Australia as a first-launch country for new medicines, ensuring that its assessment processes keep pace with rapid advances in health technology [29]. The HTA Review will run for 12 months, until December 2023, with recommendations to be implemented within 12 months (December 2024).

The French compassionate program called “Temporary Authorization for Use” (ATU) is an example where the use of drugs before their market authorization is allowed in indications without appropriate therapies and for which no clinical trials are being conducted for the intended use [30]. A major reform in 2021 introduced the need to meet the following three criteria: (1) a presumption of innovation compared with the most clinically relevant comparator; (2) an appropriate development plan; and (3) the absence of significant safety or tolerability uncertainties [31]. Since its application, most products have met all criteria (e.g., idecabtagene vicleucel, pembrolizumab, azacytidine, nivolumab, sacituzumab-govitecan).

Finally, to ensure that HTA solutions are applied within a reasonable timeframe, performance standards should be specified to set maximum total timeline targets for the drug review processes. If longer delays are encountered, immediate managed access programs for funding should be mandated while standard processes continue. Since many of these proposed solutions may be expensive, the development of a process to de-list older and less expensive medicines when newer innovative drugs are developed would allow cost reduction and increased funds for breakthrough medications. However, it is worth mentioning that new cancer drugs defined as “high-cost” by the PMPRB account for less than 1% of national health expenditure in Canada [32].

## 5. Conclusions

Canada’s public drug reimbursement timelines continue to increase, detrimentally impacting cancer patients, for whom treatment delays are particularly detrimental to their survival and QoL. Indeed, as demonstrated in a TLR identifying 56 studies, delayed access to oncology drugs shows clear clinical, economic, and QoL impacts for cancer patients. Although most results and findings of this review represented delayed access to cancer drugs in individuals from diagnosis to the beginning of treatment, the latest Canadian publications assessing process-related delays confirm clinicians’ observations that patients who would likely have benefited from new treatments are dying without having the opportunity to try them.

Initiatives should be put in place to improve the performance and speed of Canadian drug regulatory and HTA processes, especially for new cancer therapeutics. CADTH has recently proposed to introduce time-limited reimbursement recommendations to ensure timely access to promising new therapies for serious conditions where there is an unmet medical need [33]. This is a first step, but more initiatives are needed. The proposed solutions in this paper include better coordination between HTA and Canadian provinces to harmonize coverage decisions, international collaborations, information sharing, and national standards for timeliness in oncology drug access.

With Canada lagging behind other countries, it is imperative that we act quickly, as the survival and quality of life of Canadian cancer patients depend on it. Many other OECD countries have successfully recognized deficiencies in their regulatory and HTA systems and have implemented changes to rectify them. For example, the European Union Member States have acknowledged that HTA working practices and appraisals differed considerably across Europe, and the European HTA Regulation was adopted to address this situation. Recent advancements have led to breakthrough cancer therapies, and the long assessment and reimbursement processes in Canada prevent cancer patients from accessing these treatments. Many patients in Canada do not have access to a private health insurance plan, and this cannot be relied upon for access [34]. In addition, cancer patients are often older, and most seniors are covered publicly in Canada. We now live in a two-tiered reality where patients with financial means or comprehensive private drug insurance plans are receiving care unavailable to other citizens. This deplorable situation requires an urgent revolution of Canada’s regulatory and HTA systems. Canada needs to acknowledge its deficiencies and evolve to ensure timely and equitable access to new drugs for all patients—fortunately, there are numerous proven solutions at hand.

## Figures and Tables

**Table 1:** Characteristics of studies included in the TLR.

First Author, Year, Location	Objective	Study Design	Type(s) of Cancer	Types of Treatment	Outcomes Reported
Gotfrit, 2020 [14], Canada	To quantify the potential life years lost during Health Canada drug approval and funding process	Public data analysis	Lung, breast, and colorectal cancers	Drugs submitted to pCODR	OS, PFS, life years lost
Stewart, 2018 [13], Canada	To calculate life years potentially saved if selected agents were approved more rapidly	Public data analysis	Advanced malignancies	New therapies for metastatic cancers	Life years saved
Uyl-de Groot, 2020 [15], Europe	To assess the access in Europe to newly registered cancer drugs and to obtain more insight in the implications of these variations for patients	Retrospective database study	Breast cancer, gastric cancer, prostate cancer, and melanoma	Chemotherapy and targeted therapy	Life years lost
Vanderpuye-Orgle, 2022, Global [16]	To better understand the impact of any additional delays on NSCLC patients	Analysis of literature and empirical modelling	NSCLC	Chemotherapy, drugs for NSCLC	Health care costs, QALY

Abbreviations: NSCLC: non-small cell lung cancer, OS: overall survival, pCODR: pan-Canadian Oncology Drug Review, PFS: progression-free survival, QALY: quality-adjusted life year, RFS: recurrence-free survival.

## Supplementary Materials

The following supporting information can be downloaded at: https://www.mdpi.com/article/10.3390/curroncol31030110/s1, Table S1: Clinical Search Strategy; Table S2: Economic Search Strategy; Table S3: Quality of Life Search Strategy.
