# Supplementary material for: Impact of Systemic Delays for Patient Access to Oncology Drugs on Clinical, Economic, and Quality of Life Outcomes in Canada: A Call to Action
Source: Curr Oncol. 2024 Mar 11;31(3):1460–9. doi: 10.3390/curroncol31030110 (PMC10969399; doi:10.3390/curroncol31030110)
Supplement: Supplementary file 1 [file curroncol-31-00110-s001.zip › curroncol-2784585-supplementary.pdf]

Current Oncology

Bibliographic Search Strategies

PubMed (Date of search: July 21, 2022)

Table S1: Clinical Search Strategy

#	Query	Results from 21 Jul 2022
**Ovid MEDLINE(R) ALL <1946 to July 21, 2022>**		
1	cancer*.ab,ti.	2,074,376
2	Neoplasms/	479,846
3	1 or 2	2,267,933
4	Time-to-Treatment/ or treatment delay.ab,ti.	11,403
5	life year* lost.mp.	590
7	4 or 5	11,988
8	Disease-Free Survival/ or Survival/ or Progression-Free Survival/	92,716
9	mortality*.ab,ti.	905,477
10	8 or 9	989,072
11	3 and 7 and 10	452
12	limit 11 to yr="2017 -Current"	284
13	limit 12 to (editorial or letter or case report or case review or opinion) [Limit not valid in Ovid MEDLINE(R),Ovid MEDLINE(R) Daily Update,Ovid MEDLINE(R) PubMed not MEDLINE,Ovid MEDLINE(R) In-Process,Ovid MEDLINE(R) Publisher; records were retained]	3
14	12 not 13	281
15	limit 14 to (abstracts and humans)	**264**
**Pubmed (2021-2022)**		
1	(treatment delay) AND (cancer) AND (clinical impact)	**289**

Table S2: Economic Search Strategy

#	Query	Results from 25 Jul 2022
	**Ovid MEDLINE(R) ALL <1946 to July 21, 2022>**	
1	cancer*.ab,ti.	2,075,532
2	Neoplasms/	480,043
3	1 or 2	2,269,127
4	Time-to-Treatment/ or Time Factors/	1,235,064
5	Economics/ or Quality-Adjusted Life Years/	42,472
6	Health Care Costs/ or "Costs and Cost Analysis"/	92,261
7	5 or 6	131,510
8	3 and 4 and 7	514
9	limit 8 to yr="2017-Current"	142
10	limit 9 to (editorial or letter or case report or case review or opinion) [Limit not valid in Ovid MEDLINE(R),Ovid MEDLINE(R) Daily Update,Ovid MEDLINE(R) PubMed not MEDLINE,Ovid MEDLINE(R) In-Process,Ovid MEDLINE(R) Publisher; records were retained]	2
11	9 not 10	**140**
	**Pubmed (2021-2022)**	
1	((treatment delay) OR (drug delay)) AND (cancer) AND ((economic impact) OR (economic burden))	**72**

Table S3: Quality of Life Search Strategy

#	Query	Results from 25 Jul 2022
	**Ovid MEDLINE(R) ALL <1946 to July 21, 2022>**	
1	cancer*.ab,ti.	2,075,532
2	Neoplasms/	480,043
3	1 or 2	2,269,127
4	Time-to-Treatment/ or treatment delay.mp.	11,505
5	Drug Approval/ or delay* access.ab,ti.	16,991
6	4 or 5	28,468
7	Quality of Life/	248,211
8	3 and 6 and 7	111
9	limit 8 to yr="2017 -Current"	61
10	limit 9 to (editorial or letter or case report or case review or opinion) [Limit not valid in Ovid MEDLINE(R),Ovid MEDLINE(R) Daily Update,Ovid MEDLINE(R) PubMed not MEDLINE,Ovid MEDLINE(R) In-Process,Ovid MEDLINE(R) Publisher; records were retained]	4
11	9 not 10	57
12	limit 11 to (abstracts and humans)	**55**
	**Pubmed (2021-2022)**	
1	(treatment delay) AND (cancer) AND (quality of life)	**152**
